# Implicit neural representation for scalable 3D reconstruction from sparse ultrasound images

**DOI:** 10.1038/s44384-025-00018-5

**Published:** 2025-08-08

**Authors:** Tal Grutman, Mike Bismuth, Bar Glickstein, Tali Ilovitsh

**Affiliations:** 1 School of Biomedical Engineering, Tel-Aviv University, Tel Aviv-Yafo, Israel; 2 The Sagol School of Neuroscience, Tel-Aviv University, Tel Aviv-Yafo, Israel

**Keywords:** Ultrasonography, Engineering

## Abstract

Although volumetric ultrasound is limited by cost and availability of 2D arrays, 3D volumes can be reconstructed from 2D slices if transducer position is known, which is not usually the case. Even with position data, existing algorithms for reconstruction are impractical due to their discrete nature that struggles with scale. We propose a 1D array on a programmable motor for scanning and implicit neural representations for continuous reconstruction. Our network’s ability to sample at arbitrary positions was compared to classic algorithms, achieving x7.9 performance while maintaining accuracy. Based on these, a reconstruction pipeline was tested on simulated data with 93% accuracy using only 36 B-mode images. This was evaluated in-vivo to measure tumor volumes in mice, with 6.3% mean error. Our findings suggest implicit neural representations can reduce data needed to recreate volumes from 2D slices and replace interpolation methods to enable interactive analysis.

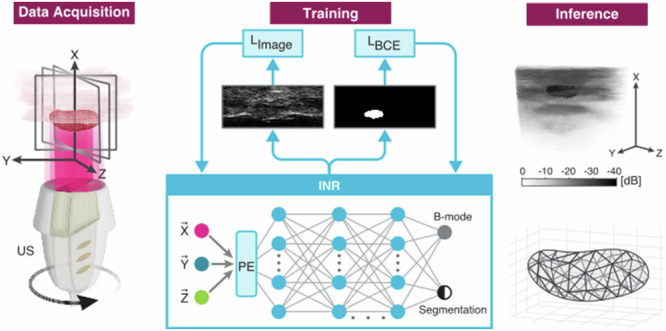

## Introduction

Ultrasound has proven itself to be a prominent imaging method thanks to its inherent advantages that include high frame rate, large penetration depth, mobility and relatively low cost^1^. In conventional ultrasound, a 1D transducer array is held in contact with the patient, and a sound wave is emitted into the body. The sound wave’s reflection is recorded and beamformed to create a 2D ultrasound image in the lateral and axial directions, representing the transducer’s width and the wave’s path into the tissue respectively. Ultrasound images are highly dependent on the transducer position and orientation (pose), as they are acquired from a 3D anatomy using a 1D array, and as a result, ultrasound imaging requires a highly skilled user with knowledge of the underlying anatomy to obtain clinically useful images. 3D ultrasound can help mitigate this challenge by removing degrees of freedom, as a wider variety of positions and orientations would capture the desired 3D anatomy.

One option for volumetric ultrasound is to use a 2D matrix array transducer such that the emitted sound wave reflection can be resolved along both the transducer’s width (lateral) and height (elevation), resulting in a 3D volume. However, the number of transducer elements in a 2D array grow parabolically with array size, and while volumetric scanners utilizing 2D matrix arrays are available^2^, they face significant technical challenges at the high frequencies and frame rates typical to ultrasound^3^, for example, due to the high rate of data transfer necessary to create a 3D volume. Consequently, existing volumetric scanners tend to be expensive and often require integration with substantial computing hardware for real-time data transfer and beamforming, diminishing some of the natural advantages of ultrasound^3^. Alternatively, knowledge of the transducer pose can be used to stitch a 3D volume from 2D slices, and the unmatched supply of 2D B-mode images in classical ultrasound makes 2D-to-3D registration a tempting solution for creating volumes. Since ultrasound images are typically recorded freehand with unspecified pose, this approach requires additional sensor inputs like inertial motor units^4,5^, optical sensors^6^, cameras^7^ or an algorithm designed with built-in knowledge of the target volume anatomy^8^, to predict the position in 3D space of a given image or set of images. Using a mechanical, software-guided motion^9^ to collect images can overcome this problem and provide precise coordinates for each 2D image, simplifying the creation of a 3D volume from the set of slices. Still, 2D B-mode images suffer from the partial volume effect, as they are generated from elements with non-zero length in the elevation direction. In contrast to volumes constructed by 2D array transducers, which can perform 3D focusing, 2D-to-3D registration can be damaged by blurring of boundaries in shapes that are asymmetrically sliced by the image plane. This is a known challenge in 2D ultrasound that can be alleviated in post-processing using geometric constraints on the 3D reconstructed volumes^10^.

Once images are collected in 2D, it is still non-trivial to represent them as a 3D volume even with known pose. Common explicit representations like voxel grids, polygon meshes and point clouds^11^ are not always scalable for real-world applications, because they are inherently discrete, imposing computation limitations on common imaging requirements. For example, doubling the resolution of a 3D voxel-grid in each axis requires x2^3^ as much memory. Interpolating the intensity of missing voxels based on nearby data is particularly difficult in 3D because the added dimension introduces numerous potential reference points for each of the new voxels. Neural radiance fields (NeRF) were introduced to overcome this^12^ by treating the volume as a continuous function of the camera pose and the image data as an implicit result of rendering an image from the volume function sampled from a particular pose. A deep neural network is trained to approximate the volume representation function, and once trained, can be sampled arbitrarily. NeRF is inherently aligned with 2D-to-3D problems, since the network is designed to learn the underlying relationship between 3D coordinates and their 2D projection in space.

In medical imaging and especially in ultrasound, implicit neural representation (INR) can be used to relax NeRF’s underlying assumptions on the volume. In INR, the network is trained so that sampling the volume yields image intensity directly without need for rendering. Algorithms based on NeRF^12–14^ and INR^10,15–19^ have reshaped the landscape of 2D-to-3D ultrasound. By taking advantage of the neural network’s continuous representation of the volume, INR can address significant challenges in 3D imaging that are generally solved with interpolation, such as filling in missing voxels or changing the resolution at which the volume is displayed. However, generating 3D images from 2D slices requires precise positioning of the 2D slices in 3D space in order to represent the volume accurately, and NeRF-based algorithms trained to represent the volume share this common need for high-quality coordinates accompanying the B-mode images. Another challenge in 2D-to-3D ultrasound is that even when a target object is present in the field-of-view, it is not trivially separable from the background. Having stitched together a set of 2D images, often the goal is to acquire the 3D shape of a particular object encapsulated within this volume. In recent years, deep neural networks have greatly improved the standards of ultrasound segmentation^20,21^, and their roles in 2D-to-3D imaging having grown to include segmentation when it is necessary. When segmentation masks of 2D frames are available, INR and NeRF can be trained to learn them alongside the B-mode images to create a continuous representation of the target object in 3D.

**Fig. 1:**
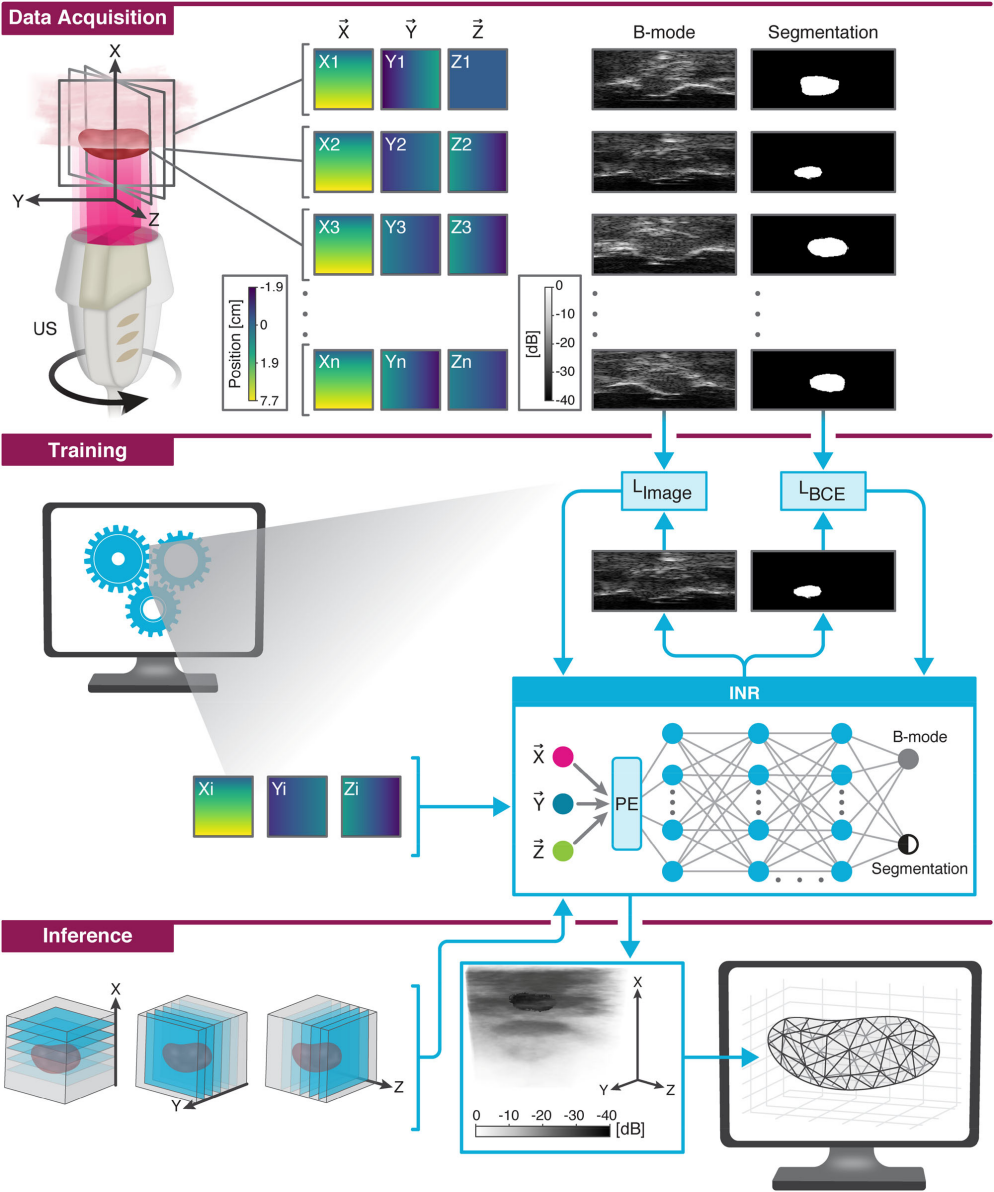
Illustration of the proposed method for implicit volume representation. In the data acquisition phase, the transducer is rotated to acquire images of the volume, and segmentation masks are extracted from each frame. During training, an INR learns to represent the volume as a mapping from image coordinates to B-mode and segmentation masks. Finally, the trained INR can freely sample the volume and provide 3D reconstruction of the object.

In our work, we use an ultrasound transducer attached to a motor and controlled programmatically, which we have used in the past to induce mechanical ablation with nanodroplets^22^. Using this setup, we create a controlled imaging protocol that precisely synchronizes between imaging and transducer movement. Captured 2D images are segmented to separate the target object from the background, and an INR network is trained on the B-mode and segmentation masks to create a continuous representation of the 3D volume (Fig. 1). Our method can be used in various volumetric ultrasound applications with static anatomy, including but not limited to tumor detection and follicle analysis.

## Results

### Volumetric imaging simulation

**Fig. 2:**
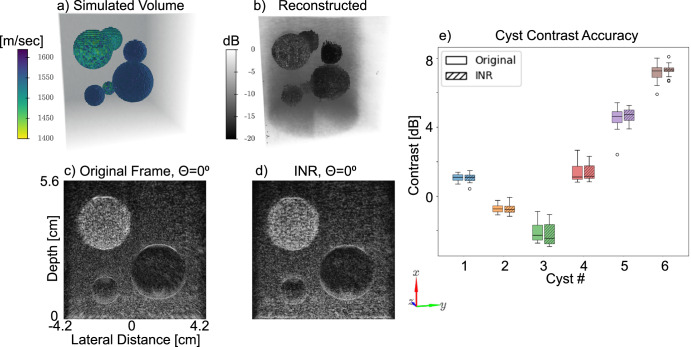
Simulation results of spherical objects with varying contrast levels. **a** Target volume representation with voxel-wise sound speed used to create contrast targets and the reconstructed volume. **b** Reconstructed INR image presented as a 3D B-mode image. Examples of a single frame from the set, at a rotation angle of 0°, of: **c** Original B-mode, and **d** INR reconstructed image. **e** Box-and-whisker plot with bars spanning the range of sphere contrast among original and reconstructed frame in the dataset from minimum contrast (bottom) to maximum (top). B-mode images displayed at 0.21 mm × 0.32 mm resolution (axial × lateral).

First, we show the ability of INR to represent images with diverse contrast. We use our simulated dataset of low-contrast spheres embedded in tissue-mimicking phantom and train an INR on the full dataset that includes 2D images of the sampled volume at 1.25° rotation intervals such that the entire 180° of the volume was scanned in 144 frames. The volume includes 6 spheres: three anechoic and three echogenic with random sphere sizes (4–15 mm in diameter) distributed randomly in the volume (Fig. 2). The volume itself is displayed with each voxel colored according to its sound speed (Fig. 2a). Each 2D image captured a cross section of the volume, hence, only 1–3 spheres were observed in individual frames at a time (Fig. 2c). The trained INR reconstruction was compared to individual frames (Fig. 2c–d), to validate the accuracy of INR reconstruction. By sampling the INR at coordinates corresponding to the entire volume at once, we created the volume image (Fig. 2b). The INR reconstruction for each sphere was evaluated on a per-frame basis by analyzing each sphere contrast for each rotation angle in the dataset as a box-and-whisker plot (Fig. 2e), where the minimum, median, and maximum of contrast are shown across all frames containing each of the spheres as the lower bar, middle bar, and top bar for each box. During rotation of the transducer, sphere size and position determined how many of the 144 total frames contained a slice of each sphere. As a result, each of the target objects appears for a different number of frames in the dataset. The average deviation of contrast between INR-generated images and originals across all spheres and frames was 0.17 dB. A two-sided *t*-test suggested that the contrast of the original frames was not statistically significantly different from the INR frames with a *p* value of 0.9.

**Fig. 3:**
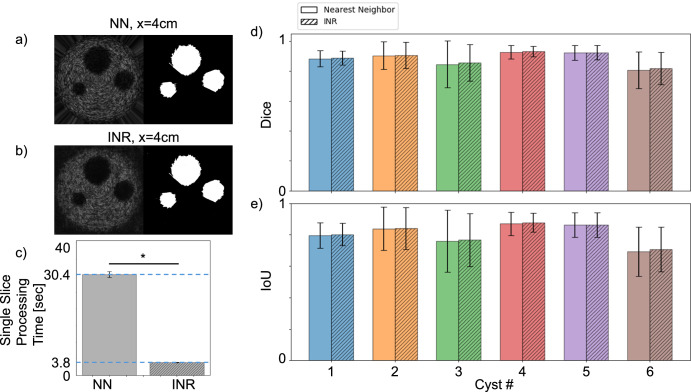
Sampling with arbitrary coordinates Comparison to nearest neighbor interpolation. Algorithm comparison of NN and INR interpolation on upsampling new views of the rotation data along the × (depth) axis. Sample outputs from each algorithm for **a** NN, and **b** INR evaluated at a depth of 4 cm. **c**–**e** Comparison metrics between NN and INR, showing the mean (bar height) and standard deviation (error bar) for each metric: **c** Single slice processing time. **d** dice coefficient, and **e** IoU. B-mode images displayed at 0.21 mm × 0.32 mm resolution (axial × lateral).

To assess the capability of INR for arbitrary sampling, the INR network is sampled in slices along the axial direction, perpendicular to the image plane used during data acquisition at ×16 the original image resolution (4096^2^) and compared to nearest neighbor interpolation (NN). A sample slice from 4 cm depth shows the B-mode and segmentation mask results of NN interpolation (Fig. 3a) and INR (Fig. 3b). The dice coefficient and IoU scores for each slice are calculated relative to the phantom model used to simulate the data (Fig. 3d, e). A one sided *t*-test was performed to evaluate whether the INR outscored NN in dice coefficient or IoU, and whether INR performed the calculation significantly faster. Although the *p*-value is insignificant for both dice coefficient (0.41) and IoU (0.43), INR completes the calculation ×7.9 faster (3.8 s for INR and 30.4 s for NN) thanks to its simplicity and reliance on GPU parallelization (Fig. 3c) resulting in a *p* value of 0 when comparing the processing time for a single 4096^2^ frame. This means that INR can recreate images and segmentation masks significantly faster than NN without a decrease in segmentation metrics.

**Fig. 4:**
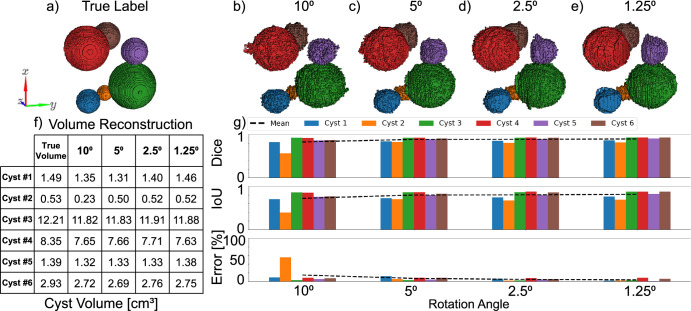
Effect of angle resolution on volume estimation Impact of rotational resolution on volume accuracy. **a** Ground truth model of individually labelled cysts. 3D segmentations produced by the INR network trained at increasingly finer rotational resolution: **b** 10°, **c** 5°, **d** 2.5°, **e** 1.5°. **f** Volume of each cyst in cm^3^. **g** Dice score, IoU, and voxel count error in % for each cyst (bars, color-coded according to their labels in (**a**–**e**) and the mean across all cysts (dotted black line) for each rotation resolution. Cysts are color-coded according to the legend visible in (**g**).

Next, we explore a volume estimation setup, in which the same volume model is used but the cysts are anechoic. We analyze the INR’s volume estimation accuracy based on the resolution of the rotation angle used in the volume sweep (Fig. 4), compared to the digital model from which the data is simulated (Fig. 4a), as well as the INR reconstruction with progressively finer rotation angles (Fig. 4b–e). The resulting volumetric estimation is summarized (Fig. 4f) and the dice coefficient, IoU, and voxel count error for each sphere in the dataset are shown, as well as the mean across spheres (dotted line) at each rotation angle (Fig. 4g). All three metrics showcase the relationship between rotation angle resolution and the size of the target volume, where the smallest sphere with a volume of 0.53 cm^3^ is notably underestimated when the rotation angle resolution is inadequate. Still, the larger volumes are reconstructed even with a rotation angle resolution of 10°, this corresponds to a dataset of just 18 frames spanning the entire volume. Although the volumetric accuracy continues to improve with finer rotation angles, an average accuracy of 93% is achieved across cysts using 5° resolution.

In addition, we tested the spatial distribution of INR errors to investigate the effect of the volume being sampled more densely near the rotation axis. We evaluated the trained subsampled INRs (Fig. 4b–d) on the full dataset of 144 frames sampled at 1.25° intervals. INR outputs of intensity and binary masks were compared to the true data sampled at each angle. For image output we calculated the mean-squared error of the pixel-wise normalized images, and for binary segmentation masks the percentage of incorrectly segmented pixels. Finally, we calculated the spearman correlation between the errors with the radial distance from the rotation axis. This gave a mean correlation of −0.03 for image intensity errors (Figure S1a) and −0.05 for binary segmentation errors (Figure S1b), suggesting that INR error is not monotonically distributed with the radial distance despite having more data points to train from near the rotation axis.

### Volume estimation of a real-world multi-object phantom

**Fig. 5:**
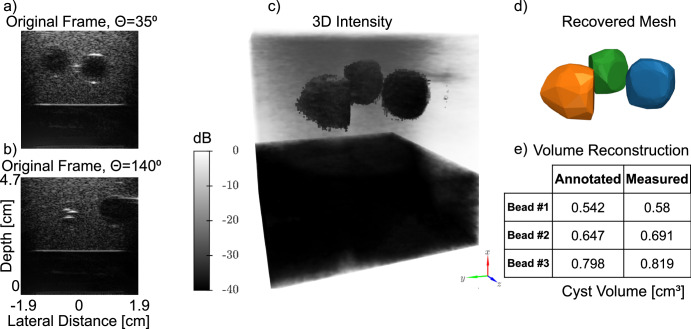
Experimental results of volume estimation in a water bead phantom 3D Volume estimation of water beads using INR reconstruction. Frames from the dataset at different rotation angles: **a***θ* = 35° and **b***θ* = 140° highlight the need for 3D to understand the composition of the volume, as anechoic water beads enter and exit the frame during scanning. **c** 3D B-mode image produced by an INR trained on this dataset, **d** Labelled meshes recovered from the INR volume. **e** Estimated volume and manual annotation. B-mode images displayed at 0.18 mm × 0.15 mm resolution (axial × lateral).

A 3D agarose-based phantom containing anechoic water beads embedded within agarose was used to test the method experimentally. The phantom contained 3 beads located triangularly at the same depth of 3.5 cm. In each 2D ultrasound image, the beads either entered or exited the frame but never appeared simultaneously during rotation of the transducer. Examples of individual 2D images at angles of 35° and 140° from the dataset of 144 are shown (Fig. 5a, b). After training, the resulting INR is sampled at the entire volume to produce the 3D intensity image (Fig. 5c). The 3D segmentation of each bead was generated and visualized as a triangle mesh (Fig. 5d), from which the volume of each bead was calculated. The recovered mesh volume was compared to the annotated volume of the beads (Fig. 5e), achieving a mean error of 5.5 ± 2.5%.

### Volume estimation of in-vivo tumors

**Fig. 6:**
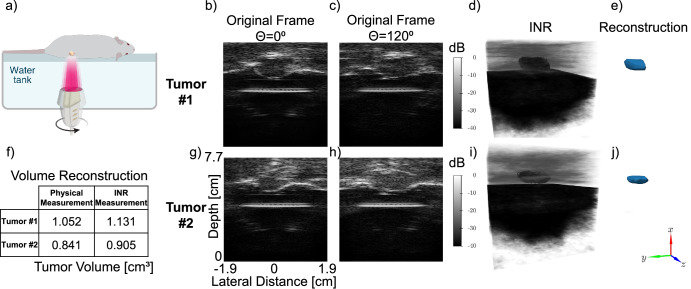
In vivo tumor volume estimation with INR reconstruction. **a** Schematic diagram of the experiment setup. Sample images from Tumor #1 at **b***θ* = 0° and **c***θ* = 120°. **d** 3D volume image reconstructed by an INR trained on Tumor #1. **e** Mesh of Tumor #1 isolated from the surrounding volume. **f** Estimated volume by analysis of the tumor mesh produced by INR compared to manual measurement. **g**–**j** Similar to (**b**–**e**) on Tumor #2: sample B-mode images at **g***θ* = 0° and **h***θ* = 120°. **i** 3D volume image and **j** reconstructed mesh of Tumor #2. B-mode images displayed at 0.3 mm × 0.15 mm resolution (axial × lateral).

Lastly, we performed in-vivo scans on five tumor-bearing mice to reconstruct their 3D volumetric structure and calculate tumor volumes. Imaging was performed with the mice lying on their side on top of an agar spacer above the water tank (Fig. 6a). For the representative two tumors in Fig. 6, a pair of 2D ultrasound images at angles of 0° and 120° are presented (Fig. 6b, c, g, h). These images highlight the view-dependent morphology of the tumors. After training an INR on each of these datasets, the 3D volume images are reconstructed (Fig. 6d, i), and tumor meshes are recovered (Fig. 6e, j). Finally, the tumor volumes encompassed by the meshes are compared to our own measurements with an error of 6.8 ± 1.5% (Fig. 6f).

The experiment shown in Fig. 6 was repeated with five different mice. Table 1 compares the INR’s approximated volume for each tumor with the elliptical approximation annotated from the B-mode images.

**Table 1:** Tumor volume estimation in vivo using manual physical measurements and volumes estimated from the tumor mesh produced by INR across five mice

	Physical measurement [cm^3^]	INR measurement [cm^3^]	Error [%]
Tumor #1	1.052	1.131	7.44
Tumor #2	0.841	0.905	7.53
Tumor #3	0.857	0.821	4.18
Tumor #4	1.023	0.947	7.38
Tumor #5	0.395	0.365	7.56

## Discussion

The future of deep learning in ultrasound is promising thanks to ultrasound’s high frame rate, mobility, low cost and safety. These advantages enable the collection of large-scale datasets to train deep neural networks that can overcome the low SNR of ultrasound to create valuable applications in various fields like holography^23^, beamforming^24^, segmentation^20,21^, speed-of-sound estimation^25^, super-resolution^26^, and more. Many deep learning and classical applications^27–29^ in ultrasound can be extended to 3D given the right data. Our work, which is also a deep learning application, fits into this ecosystem by helping to bridge this gap as the 2D-to-3D component in the pipeline of downstream tasks utilizing neural networks.

To this end, it was important to show that INR is capable of representing diverse ultrasound images accurately and maintains the contrast of various targets so that downstream tasks can rely on realistic views of the data. We showed that the INR successfully maintained contrast up to 0.17 dB and can be relied on for further applications in low-contrast scenarios. INR does this despite representing the data in a compressed manner: the size of the INR model’s weights (2.9 MB) is almost ×3 smaller than the images used to create it (8.4 MB). INR is also useful for arbitrarily rendering views of the volume. Sampling the volume at MxN points to create an MxN B-mode image requires a single forward pass of the neural network, which is a highly parallelizable GPU operation. On the other hand, the nearest neighbor algorithm used in this paper^30^ is based on k-d trees which complete the interpolation query in O(MNlog(D))^31^, where D is the total number of pixels in the dataset. In our dataset of only 144 frames at 256^2^ resolution, D was relatively small (~10^8^), but still the INR significantly outperformed the k-d tree computation. This highlights a well-known property of neural networks: the computational complexity at inference time does not rise with the size of the dataset^32^, and this advantage is expected to continue to scale up as the dataset size increases. Nonetheless, parallelizable GPU interpolation is an area of active research^33^ with important implications for 2D-to-3D ultrasound, though GPU-based interpolation algorithms that require iterating over data points like the one used here will still be disadvantaged compared to INR as dataset size grows.

This work focused on volume estimation as the downstream task, studying seven scenes: two simulated phantoms with identically placed volumetric objects but differing acoustic properties, one in-silico phantom, and five tumors in vivo in mice. While we did use an elliptical approximation to calculate ground-truth tumor volumes, the tumors were arbitrarily shaped, showing that our method can handle an assortment of datasets, shapes, and sizes. Given the versatility of our data, ranging from simulations to in vivo tumor data, we can assume that our algorithm is robust and could perform in a similar manner on a general dataset that was acquired using our rotating acquisition setup. Two interesting points that rose from these experiments was that the distribution of error is not correlated with distance from the transducer. On the other hand, the cylindrical geometry of the setup leads to a linearly growing voxel size as a function of distance from the rotation axis. This means that although the INR does not induce greater error when sampled farther from the rotation axis, the resolution of a captured volume will decrease near the end of the field-of-view, and it is preferable to center the objects if possible.

The anechoic cyst simulation was designed to resemble follicle detection typical to IVF ultrasound^34^. In this case, minimizing the number of frames in the dataset not only shortens the scan but also reduces the training time required for INR. The cysts in this simulation were reconstructed with a mean accuracy of 93% using a dataset of just 36 frames, but it was also shown that the volume can be sampled with finer rotation angles when scan time is less important than accuracy. Minimizing the number of collected frames is also advantageous because it reduces the training time needed to create an INR, however due to the cylindrical nature of the imaging setup, the voxel size grows linearly with both the rotation angle and the radial distance from the rotation axis. An optimal setup should aim to center the rotation axis and the target object while utilizing as fine of a rotation angle as possible. Interestingly, we did not see that voxels closer to the rotational axis were reconstructed with greater accuracy in our simulation.

In our tissue-mimicking phantom and in-vivo experiments, we addressed some of the difficulties of 2D-to-3D pipelines in the real world. Many image registration techniques are feature-based^35^ and as a result are confounded by multiple, similar looking objects that enter and exit the frame like the gel bead phantom described here. This challenge is resolved by the rotating transducer as the true coordinates are given, and all that is left is to stitch together an accurate representation of the 2D slices. In the in-vivo experiments, we presented a real scenario where volume estimation is key, generating a 3D model of the tumors despite their complex morphology. Other works have shown that such methods can work for other geometries such as the aorta^19^ and spinal cord^36,37^.

Still there are many available options to extend this work further. Both the phantom and in-vivo experiments suffered errors in volume estimation, with a volume error of 6.3% on average. Part of the reason for this is that all our annotations were done semi-manually due to our reliance on MedSAM, but we hypothesize that better results could be achieved with more precise labels, as seen in the simulated experiments. More work needs to be done to investigate the downstream impact of segmentation quality on volumetric accuracy, accounting for different segmentation algorithms as well as post-processing of the extracted geometry.

We chose to use MedSAM as the segmentation component because it was pre-trained on diverse types of ultrasound segmentation tasks, enabling us to semi-manually segment our various datasets. Notably, the authors of MedSAM reported that MedSAM outperformed classic medical segmentation networks in tumor detection, however, a full volume analysis pipeline for a particular downstream task could use a fully automated segmentation component that is trained for the appropriate type of target objects. This would improve segmentation accuracy leading to more precise 3D volumes given that MedSAM is not ultrasound-dedicated and can certainly be improved upon for more specific use-cases. We note that MedSAM especially struggled in frames where an object was gradually exiting the field-of-view, accounting for the flattened sides of spheres visible in Figs. 4e and 5d.

The volume scan we described here was based on rotation, but the key contribution of the mechanical component was concrete knowledge of the transducer’s position in space. Other applications using mechanical and robotic control of the transducer for various applications such as super-resolution^28^, registration to other imaging modalities, and more^38^ can also benefit from using INR as their 2D-to-3D component.

Our implementation is an early step in harnessing absolute coordinate systems based on mechanical motion to create implicit representations, however volumetric data can benefit from more complex representations based on Ultra-NeRF^13,14^ which could provide valuable anatomical insights into the volume.

## Methods

### Implicit neural representation of ultrasound volumes

In classical computer vision, natural images describe a 2D projection of a 3D scene, and the NeRF algorithm learns a 3D function that implicitly describes the available image data, each of which is a projection of a volume from a different viewpoint. However, in ultrasound, the image plane directly intersects with the volume, and B-mode intensity of a pixel is directly representative of the volume at its coordinate^13^. Optionally, the network can also be trained to predict a binary segmentation mask of the volume. The resulting INR model learns to approximate a continuous function *F* that can be sampled at 3D positions to retrieve B-mode intensity *I* alongside the segmentation mask *S*:

1 $$F\left(x,y,z\right)=\left(I,S\right)$$

Once an INR model is fitted to the volume, we can take advantage of the continuous implicit representation for various tasks. For example, by up-sampling the coordinates (*x*, *y*, *z*), the network output will be an up-sampled version of the original image. We can also choose the (*x*, *y*, *z*) vectors in ways that were not originally present in the dataset to synthesize new views of the data that the transducer did not necessarily image. Such tasks, normally completed with various interpolation schemes that require complex data structures to process in 3D, are simple function evaluations to an INR. It is important to note that NeRF and INR models are not general-purpose models like those commonly used for segmentation and classification. Each instance of our INR model is separately trained on a specific dataset, in a one-to-one relationship between a single dataset and INR model.

### Imaging setup

Our rotating transducer setup is based on a 28.2 mm-wide, 128-element phased array with a pitch of 0.22 mm, elevation aperture of 13.5 mm, and center frequency of 3.5 MHz (IP104, Sonic Concepts, Sonic Concepts, Bothell, WA, USA)^39^ assembled on a motorized rotary (RTY-IP100, Sonic Concepts) and controlled by a programmable ultrasound system (Vantage 256, Verasonics Inc., Redmond, WA, USA). This rotating transducer setup was used across all experiments. In the case of the in-silico and in-vivo experiments, the diameter of objects was manually annotated on the B-mode images.

### Simulated data

In the first set of experiments, this transducer was simulated using the Python wrapper for k-Wave^40,41^. The transducer’s operating frequency was set to 3 MHz and a field-of-view of 5.5 cm in the axial direction and 8.2 cm in the lateral direction was created using a virtual grid of size 280 × 412 × 88. Six spheres with random radius in the range of [4, 15] mm were randomly placed in a volume of soft-tissue. The volume was sampled at 1.25° rotation intervals such that the entire 180° of the volume was scanned in 144 frames. Five plane waves were simulated at each rotation angle, each steered at angles linearly spaced along [−5°, 5°]. Raw RF data was stored for post-processing. Two experiments were created by modifying the acoustic parameters of the spheres. In the first experiment spheres were simulated to be weak contrast targets in the range of [−2, 8] dB with respect to the background to test the INR’s accuracy in representation of intensity. A sound speed of 1580 m/s was used in dark spheres and 1480 m/s in bright targets. Sphere brightness was induced by varying the scattering coefficient between 0.2 in the brightest target to 0 in the darkest. The background received a scattering coefficient of 0.01 and sound speed of 1540 m/s to resemble soft tissue. In the second experiment aimed at estimating volume quality, the same volume was used but the targets were homogenous and anechoic.

### Imaging experiments

In the phantom and in-vivo studies, the transducer setup was submerged in a water tank filled with degassed water. The transducer was located at the bottom of the tank so that the positive axial direction pointed upwards to the imaging target located above it (Fig. 1). The transducer was operated by the programmable ultrasound system using an imaging script written in MATLAB (Mathworks, Natick, MA). The same imaging sequence described in the simulation acquisition was used to capture plane wave RF data and store it for offline processing. In both studies, the volume was sampled at 1.25° rotation intervals to produce a dataset of 144 frames in ~1 min, which was deemed fast enough for our research purposes. We did not attempt to optimize the volumetric sweep time but hypothesize that it could be improved for specific applications.

### Tissue-mimicking phantom

A custom 9 × 6 × 3 cm^3^ rectangular mold was filled with 250 mL of degassed water with 2.5 g agarose powder (Thermo Fisher Scientific Inc, Waltham, MA) and 1.5 g of Silicon carbide (Sigma-Aldrich, St. Louis, MO) which acted as scatterers. Scatterers were prevented from sinking to the bottom of the phantom by submerging a magnetic rod in the solution and inducing rotation of the rod with a magnetic field until the solution was brought to room temperature. Next, the solution was poured into the phantom mold, and while solidifying, 3 water gel beads (UPC: 786194299504, Made Top), soaked in water for 30 min with slight variation between them to create size differences, were placed together at the same depth of 3.5 cm from the transducer but spaced evenly in a triangle shape so that the rotating transducer aligned to the center of the triangle could not image all three beads simultaneously. The transducer was placed in a degassed water tank with the phantom submerged in the bath directly above it so that by rotating the transducer different views of the beads could be captured. An imaging field-of-view of 4.7 cm in the axial direction and 3.8 cm in the lateral direction was acquired at each rotation angle. Gel beads presumably continued to grow throughout imaging as they are highly water absorbent, so their diameters were measured by annotating the B-mode images acquired from this experiment, resulting in diameters of 4.8 mm, 5.1 mm, and 6.9 mm. Due to the perfectly spherical shape of the beads, ground truth sphere volume was calculated using the sphere volume equation based on these diameters and compared to the volume of the convex hull bounding the INR’s segmentation prediction, which was used in similar works to ours^19^.

### In-vivo experiments

Finally, we tested our method in vivo on five tumor-bearing mice. Animal-related procedures were conducted in accordance with the guidelines provided by the Institutional Animal Research Ethical Committee (approval TAU-MD-IL-2407-154–5). We used Met-1^42^ mouse breast carcinoma cells cultured in Dulbecco modified Eagle medium (DMEM, high glucose, supplemented with 10% v/v fetal bovine serum, 1% v/v penicillin–streptomycin and 0.11 g/L sodium pyruvate) at 37 °C in a humidified 5% CO_2_ incubator until about 85% confluency on the day of the injection. We collected cells by dissociation with TrypLE Express and resuspended at 1 × 10^6^ cells in 25 μL PBS+/+ for bilateral subcutaneous injection into #4 inguinal mammary fat pad of injected female FVB/NHanHsd mice (Envigo, Jerusalem, Israel). Before imaging, anesthesia was induced with 2% isoflurane in ambient air (180 mL/min) and the area in proximity of the tumor was shaved. Fur was removed with a depilatory cream to improve coupling along with ultrasound gel. Mice were placed on their side on an agar spacer prepared similarly to the one in the phantom study, at the top of the filled water tank. Unlike the phantom experiments, this meant that imaging needed to pass through the entire depth of the water tank to reach the target, and as a result the axial field-of-view was enlarged to 7.7 cm. The lateral field-of-view remained the same at 3.8 cm.

Images were collected at 35 days after cell injection, and the tumor size was measured in each direction using the B-mode images from the 3.5 MHz rotating transducer. As the tumors were visibly elliptical to the eye, we calculated the ground-truth tumor volume as the volume of an ellipse given the measured diameters in each direction, and compared to the volume of the convex hull bounding the INR segmentation prediction. The various datasets used to test our method and described above are summarized in Table 2.

**Table 2:** Summary of datasets used to showcase the properties of INR in 2D-to-3D ultrasound.

Dataset	Source	Number of Frames	Objects
Contrast simulation phantom	k-wave simulation	144	6
Follicle simulation phantom	k-wave simulation	18–144	6
Gel beads phantom	In-silico lab experiment	144	3
Mouse tumor #1	In-vivo lab experiment	144	1
Mouse tumor #2	In-vivo lab experiment	144	1
Mouse tumor #3	In-vivo lab experiment	144	1
Mouse tumor #4	In-vivo lab experiment	144	1
Mouse tumor #5	In-vivo lab experiment	144	1

### Data processing and INR implementation

After data collection for a given volume, images were beamformed with a custom CUDA^43^ library to 256^2^ resolution and a dynamic range of 20 dB for simulated data and 40 dB for phantom and in-vivo data. Segmentation masks were created semi-automatically using MedSAM^44^ to isolate the interest region from the background in each rotated image. An INR was trained using PyTorch^45^ on an NVIDIA RTX 3080 GPU (NVIDIA Corporation, Santa Clara, CA) with 10GB of GPU memory. Input coordinates to INR models employed positional encoding and sinusoidal representation network (SIREN) activations^46^ to encourage the models to learn high-frequency information^46^. Each 3D coordinate *x*_i_ in (*x*, *y*, *z*) was encoded at a logarithmic harmonic scale:

2 $$\begin{array}{c}{PE}\left({x}_{i}\right)=({x}_{i},\sin \left({k}_{o}{x}_{i}\right),\cos \left({k}_{o}{x}_{i}\right),\ldots ,\sin \left({k}_{j+1}{x}_{i}\right),\cos \left({k}_{j+1}{x}_{i}\right),\ldots ,\sin \left({k}_{N}{x}_{i}\right),\cos \left({k}_{N}{x}_{i}\right))\\ {k}_{j}={2}^{j};0\le j\le N\end{array}$$

This was the input to our model which was composed of a 10 layer multi-layer perceptron. Each layer had a width of 256, with skip connections every other layer and SIREN activations. Since positional encoding guides the network to finer resolution of volume representation, we empirically chose to use *N* = 15 for the positional encoding, at which point the output images created by the INR visibly showed speckle patterns similar to those in the original images. This gave the network input a shape of 93. The final layer produced an output of size 2 for each input position. Thus, a Bx3 batch of positions samples the network to produce a Bx2 vector of intensity and segmentation mask at each of the positions (Fig. 1).

During training of an INR on a particular volume, images, segmentation masks, and their corresponding coordinates were sampled from the available rotation angles, and the INR model was trained to replicate the image and binary mask for a given set of coordinates. We used the structural similarity index measure (SSIM) as the loss metric for image reconstruction (*L*_image_, Fig. 1) which has been used previously in other works of implicit neural representation^47^:

3 $${L}_{{image}}=\frac{(2{\mu }_{x}{\mu }_{y}+{k}_{1})(2{\sigma }_{{xy}}+{k}_{2})}{({\mu }_{x}^{2}+{\mu }_{y}^{2}+{k}_{1})({\sigma }_{x}^{2}+{\sigma }_{x}^{2}+{k}_{2})}$$

Where *µ*_x_, *σ*_x_ are the local mean and standard deviation of the image *x* and σ_xy_ is the local covariance of images *x*, *y*.

For binary segmentation, the loss between our prediction *x* and the ground truth mask *y* used binary cross-entropy together with the dice score and penalized the INR pixel-wise for false positives (*L*_BCE_, Fig. 1):

4 $${L}_{{BCE}}\left(x,y\right)=y\log \left(x\right)+\left(1-y\right)\log \left(1-x\right)+1-2\frac{\left|x\cap y\right|}{\left|x\right|+\left|y\right|}+\left|x\cap (1-y)\right|$$

We trained with the Adam optimizer^48^ for 500 epochs per volume, sped up using grokfast^49^ to achieve peak performance in minimal training epochs. The learning rate was initially set at 1 × 10^−3^ with a cosine annealing schedule that reached a minimum of 1 × 10^−6^. After training, the entire volume was sampled at once as a voxel grid at the desired resolution, and objects in the volume were labelled using cc3d^50^ before being converted to a triangle mesh wrapping the convex hull of the voxels. Once trained, INR was compared to voxel-wise nearest neighbor to assess its capabilities in 3D imaging^16,36^. We initially experimented with other interpolation algorithms like bilinear interpolation but found that the processing time was too long (on order of minutes) even for our modest dataset.

### Image similarity metrics

In experiments using simulated data, the INR can be compared to the true model or original image data from which the data is simulated to evaluate the model’s ability to capture the scene accurately. In the contrast simulation, the contrast between the i^th^ contrast target 0 ≤ *i* < *N* and the background in dB is compared between original images and INR-generated using the formula:

5 $${{Contrast}}_{i}[{dB}]={\mu }_{i}-{\mu }_{{background}}$$

where *μ* is the mean intensity in a particular region in dB. In the case of volume estimation, the binary segmentation mask predicted by INR for the *i*th object *ŷ*_i_, is compared to the true segmentation mask *y*_i_ using the dice coefficient and intersection-over-union (IoU):

6 $${{Dice\; Coefficient}}_{i}=2\frac{{y}_{i}\cap {\hat{y}}_{i}}{\sum {y}_{i}+\sum {\hat{y}}_{i}}$$

7 $${{IoU}}_{i}=\frac{{y}_{i}\cap {\hat{y}}_{i}}{\sum {y}_{i}\cup {\hat{y}}_{i}}$$

In addition, we calculate the final volumetric error in %, which indicates the discrepancy in estimation of a target volume *V̂*_i_ compared to the actual volume *V*_i_:

8 $${E}_{i}[ \% ]=\frac{\left|{V}_{i}-{\hat{V}}_{i}\right|}{{V}_{i}}$$

The volume estimation metrics described in Eqs. (6–8) are all in the range [0, 1]. It is desirable to maximize the dice coefficient and IoU while minimizing the relative volume error.

## Supplementary information

Supplementary information

## Data Availability

The datasets used in this study are available from the corresponding author on reasonable request.

## Code availability

The code used in this study is available from the corresponding author on reasonable request.
