# Supplementary material for: Implicit neural representation for scalable 3D reconstruction from sparse ultrasound images
Source: NPJ Acoust. 2025 Aug 8;1(1):14. doi: 10.1038/s44384-025-00018-5 (PMC12334353; doi:10.1038/s44384-025-00018-5)
Supplement: Supplementary file 1 — Supplementary information [file 44384_2025_18_MOESM1_ESM.pdf]

Supplementary Information

Implicit Neural Representation for Scalable 3D Reconstruction from Sparse Ultrasound Images

Tal Grutman[1], Mike Bismuth[1], Bar Glickstein[1], Tali Ilovitsh[1,2]

[1] School of Biomedical Engineering, Tel Aviv University, Tel Aviv 6997801, Israel
[2] The Sagol School of Neuroscience, Tel Aviv University, Tel Aviv 6997801, Israel.

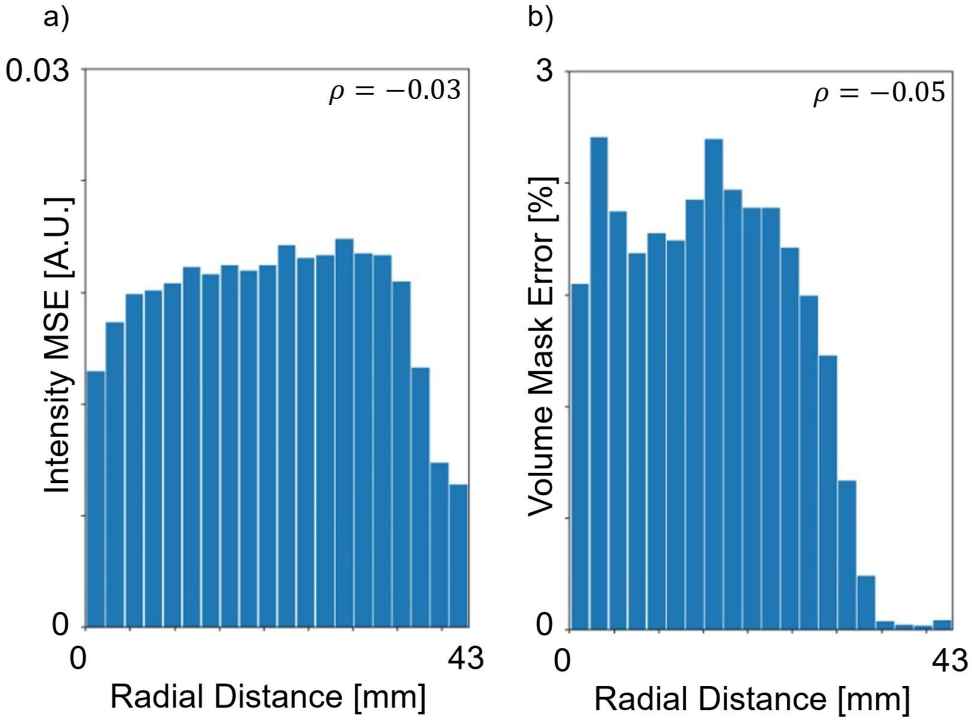

Figure S1. Radial distribution of INR error across INRs trained on partial training data. The spearman correlation coefficient ρ between the radial distance and a) error in intensity and b) error in volume mask is provided.
